# The Cast Inlay

**Published:** 1907-12-15

**Authors:** W. H. Taggart

**Affiliations:** Chicago


### THE CAST INLAY.

BY W. H. TAGGART, D. D. S., CHICAGO.

It seems to me that an era of almost painless dentistry is coming. I say almost painless because it is not absolutely so. As long as patients are human they will suffer pain whether they are hurt or not. At the same time, I feel that this process will make it possible for men of ordinary ability to achieve the same results as the high grade men. It is not only going to make the man of ordinary ability do the work which is now done by the high grade men, but it will be possible for high grade men to do work which they never dreamed of before. It is simply a process of mechanics. In the casting or molding of a piece of metal which is to fit any place, in other lines of business it is only necessary to have an approximate fit; they expect to adjust it to its place. With iron, if it shrinks and the cast is too large, the pattern is changed, so that the pattern does not represent the size of the finished casting. In that way they see-saw back and forth till they finally get a pattern which, when molded and reproduced in iron or any other material the shrinkage which takes place is compensated for by the amount of extra material in the pattern. Now, in our own line of work we need an exactness that no other line of work requires. The highest grade watch in the world today is not as accurate as ought to be a gold inlay. We all use the remark "as fine as a watch," but a watch is only fine by comparison. If a monster locomotive had as much lost motion in it as a watch has, it would tear itself to pieces, and the locomotive today is a finer piece of work mechanically than the finest watch made. The exactness of fit necessary in our own line of work makes this system, it seems to me, almost ideal. We do not dare to have a pattern which has to be altered in outline. The surface against which an inlay is to fit is so exceedingly irregular that we could not compensate for it by an addition to the pattern. We must have something that fits after it is molded without any further adjustment except the outside polish.

In the process of filling teeth with gold inlays, it has been a question of burnishing the matrix to the seat. Cavity preparation is practically the same as in any other line of gold inlay work. The matrix was fitted more or less properly, depending on the skill of the dentist, but after he had the matrix burnished to its seat he really felt in his heart that the only place it fitted was the margins he had burnished. If after these margins are burnished and the filling is put to its seat, there is occasion to do any grinding to correct a faulty articulation, what happens to the best inlay? You will immediately grind into the territory of the cement, showing that the surface which is in contact with the tooth proper does not come in the same close adaptation to all the walls of the cavity as the margins did. With the molding method a perfect fit may be made throughout the entire area to the cavity walls. The most ordinary one of you can make a fit which will be equal to the best that can be done by anyone, but the artistic end of it will depend upon your own artistic sense. The first process is to make a wax inlay in the cavity. The wax is easily shaped and formed to the proper contour and occlusion, and after this has been done, with as much artistic ability as you may, the balance of the work is merely a willingness to attend to detail. It is not a question of the personal equation at all, but simply one of mechanics.

I have felt for a good many years that there should be some improvement in inlay work, that we were almost to the limit, because there has been no improvement made in gold inlay work in the last ten or twelve years, except in the matter of technique. For that reason I have felt that we must go outside of the matrix and get some entirely new idea.

I will try now to demonstrate this process.

The preparation of the cavity is the same as in any other gold inlay method, and just here I would say that a steel tool has no business in a cavity which needs an inlay. I say that after a great deal of experience. You can take any of the carborundum wheels and with a coarse file, file them to any shape you may desire and in that way make a tool which will be very much more comfortable to the patient and with none of the danger of jumping over the cavity margin. The work is rapid and the likelihood of making unnecessary little undercuts which is apt to happen with a bur is avoided when you use the carborundum stones to prepare the cavity.

When the cavity is ready a piece of wax, specially prepared is forced into the moist cavity. The patient is then told to bite into the wax and chew on it to give the proper occlusion and articulation, wearing down the high points of the wax which otherwise would have to be ground from the filling. I then take an instrument no less sharp than an ordinary gum lancet, and trim off the excess of wax that hangs over the margins. After trimming I raise it out of the cavity while it is still plastic and unseat it, and put it back and raise it up a second time to be sure it goes in and out of the cavity without dragging, and have the patient bite on it again. That gives me a chance to correct any faulty articulation it may have. Then with burnishers I commence to smear the wax toward the margins, not allowing the instrument to stick into the wax or mutilate it. That continual wiping toward the margins of the soft plastic material such as this wax is, will give you a beautiful fih After it has been approximately trimmed, I cool it off with a few drops of water. If it gets a little plastic in the mouth all that is necessary to bring it back to a workable condition is a few drops of cold water. After it is lifted from the cavity I hold it under an ordinary hydrant faucet, letting the water run on it for some moments. It soon assumes about the same hardness as modeling compound, and by using an exceedingly sharp knife it is possible to carve the wax into any form you wish. Then carry it back to the cavity and gently push it into its place, and burnish the margins again. Take a piece of ordinary linen tape, put some vaseline on it and pass it between the teeth as you would in polishing a gold filling. By just rubbing it without any pressure you will smear it to the margins without distorting your proximal contact, and the vaseline keeps the wax from sticking to it. After rubbing that way a short time you get a highly polished surface and when the inlay is finished, you will have very little grinding to do. Take a piece of cotton about as big as your little finger, and put that in vaseline with tweezers, and wipe the surface of the inlay. Do not mistake the shining surface that the vaseline itself gives it, but keep on rubbing, and in a few minutes you will have an absolutely polished surface, which will not distort any of the cusp lines. By doing this in an artistic way, and getting the wax as smooth as you can, there is little to do on the finished inlay. Chill this last time very thoroughly and then lift it out of the cavity.

This little wire I hold in my hand is about 1-16 of an inch in diameter and $\frac{3}{4}$ of an inch long, and is called the sprue wire. I warm the very tip of it, holding it in my fingers so as not to overheat it. I make it just warm enough so the end will stick to the wax. I have the wax inlay on the end of the wire, and this wire forms the hole into which the gold is subsequently poured. The wire is put into any part of the filling which would not be dangerous to the shape or artistic lines that you have worked out.

The wax inlay on the wire is ready to be covered with investing compound. One of the greatest difficulties I had in working out this process was to get rid of air bubbles. To overcome that, I take a small quantity of investment compound no larger, say, than half a pea, and put it on the cavity side of the inlay, and then coax it ahead of an instrument, being sure to watch it so that no air bubble gets ahead of it. The vulnerable points are down in the corners that would easily confine a little air bubble. My friend, Dr. Perry, is responsible for the remark that to handle plaster properly, you should put it where you don't want it, and then push it where you do want it. That is a very good rule here.

After a generous mass of investment material has been placed around the wax it is put into a flask which is a section of a brass tube cut off, and is about one inch or one and one-quarter inches long, and I have yet to find, with one exception, any investing material in the market that I can fill that tube with and get thoroughly dry after melting the wax without the investing material sliding out of the tube. I had to find an investing material which would not do that and at the same time would have a smooth surface to cast against.

After the investment is hard in the flask the wire is pulled out leaving a hole the size of the wire leading down to the inlay. A scooped out place on the surface has been formed by the top of the flask which makes a little crucible in which to melt the gold. That crucible is formed there each time by the flask cover. When the gold is put into the crucible and melted you might think it would drop down into that hole, but it does not. The spheroidal tendency of the melted mass keeps it rolling on itself, and the fact that air is inside of the cavity prevents the gold from dropping down into the hole. The flask is put over a Bunsen flame and that melts the wax and it is absorbed into the investment and disappears.

It leaves absolutely no residue. It has been filtered three times so as to get rid of any foreign substance that might be in it.

The gold is placed in the crucible and melted with the nitrous-oxide flame into a thorough bubbling or boiling state, and then with one movement of a lever the flame is turned off and a pressure of from 15 to 25 pounds nitrous-oxide, thrown on the gold from the cylinder so as to force it into the space left by the melted wax. This results in an exact reproduction of the wax inlay in gold, and the actual time consumed in the entire process, leaving out the necessary time to wait for the investment to harden, is about thirty minutes.

The resultant product is an inlay which precisely fits the cavity and which requires no further finishing except to polish the exposed surface. Any karat gold may be used and I have some specimens of work which have been molded out of clasp gold. We have never been able to use clasp gold in dental work except for flat clasps for the reason that it was too hard and unyielding, but this process makes it possible to melt it and mold it.

In conclusion, this process of making inlays must appeal to any operator when he sees the perfection of results. It cannot be ignored by the older men because of the greater ease with which perfect work may be accomplished and the lessened strain on the operator, and it cannot be overlooked by the younger men because they have a lifetime of practice ahead of them and must take advantage of every method which renders their work more perfect and relieves them of the tension of difficult operations.—November, 1907 *Review*.

